# Destiny

**Published:** 1892-07

**Authors:** 


### DESTINY.

An intelligent person of ready wit is selected for the Oracle, and sent out of the room for a few minutes. The rest of the company, seated in a semi-circle, whisper each to each, a person's name (preferable of one present), to the left hand neighbor, and a sentiment to the right hand neighbor—ladies giving gentlemen's names and the gentlemen ladies' names. The Oracle is then called in and takes his seat, and begins an imaginative narrative suited to the circumstances and to the assembly before him—the more brilliant his part the more interesting the proceedings.

Suppose, for instance, he knows a pleasantry about any two persons present, and he remarks, beginning with the first person seated on his left, whom we will call Miss A: "What did you, Miss A, say was the name of the gentleman you heard so passionately pleading with a fair damsel for a token of affection, the other evening, at such and such a place?" (She then gives you name previously whispered to her.) It may not be the person about whom it would be true; but the hit will be nearly as good and not a few present may appreciate its full force. What is more, promiscuous blushes may reveal unsuspected developments in other directions. The Oracle then continues and asks Miss A again : "And what was the tender and expressive sentiment that he swore to be his watchword and guiding talisman?" (She here repeats the sentiment previously whispered to her.) It may or may not suit and the awkward or appropriate association will generally cause innocent confusion or merriment. The Oracle then proceeds to player B. In this way, by bringing in matter from society, politics, art, literature and whatever other fields suggest appropriate material, a very superior diversion can be enjoyed.—*Parlor Games.*

